# A eureka moment – using coalface data for discovery on TB disease and financial relief

**DOI:** 10.5588/ijtldopen.25.0550

**Published:** 2026-02-11

**Authors:** K. Watts, K. Dale, J.T. Denholm

**Affiliations:** 1 Victorian Tuberculosis Program, Royal Melbourne Hospital, Melbourne, Australia;; 2 Department of Social Work, University of Melbourne, Melbourne, Australia;; 3 Public Health, Department of Health, Victoria, Australia;; 4 Department of Infectious Diseases, University of Melbourne, Melbourne, Australia.

**Keywords:** tuberculosis, Victoria, Australia, structural inequities, social protection payments

## Abstract

**BACKGROUND:**

Although Australia maintains a low incidence of TB, elimination remains elusive. To support program delivery and evaluation, intervention fidelity, trends and patterns in the provision of financial relief were explored. Insights for practitioners and funders inform decision-making.

**METHODS:**

Data and process mining approaches were used to explore the dispersal of vouchers and small grants processed through the Victorian Tuberculosis Program. Data sources identified, curated, and analysed included financial relief dispersal records and public health documentation (sociodemographic, TB, and health care access characteristics). Queries and analysis were shaped through practitioner consultation.

**RESULTS:**

Of 3,811 TB events, 15% received financial relief during treatment. Relief recipients were younger, more often male, and more likely to have pulmonary disease and cavitation. Twice as likely to be part of whole genome sequencing-defined transmission clusters, recipients also experienced more treatment interruptions. Practitioner insights revealed that financial relief functioned as a response to hardship and as an enabler or incentive to support engagement with care.

**CONCLUSION:**

The observed patterns and trends suggest an intersection between TB vulnerability and financial relief. Low-incidence, high-income settings likely benefit from financial relief to mitigate structural inequities. Further research should detail the nature of financial hardship, co-occurring indicators of TB, and their proximity to TB care outcomes.

Although TB incidence and transmission rates are enviably low in Australia, the lofty goal of elimination remains elusive, and TB continues to be a public health concern. Incidence and mortality rates have been largely unchanged for decades.^1,2^ For those affected, the burden of TB is often deeply personal, with enduring biological and psychosocial impacts – consequences that disproportionately affect the marginalised. Despite Australia's universal health insurance scheme, private health insurance options, and public hospital funding, out-of-pocket healthcare costs remain relatively high.^3^ And although TB care is provided free of charge, economic and health inequities can intersect, impacting access to TB diagnosis, treatment and recovery. Free of charge does not mean that TB treatment is free of financial hardship.^4^ In 2023–2024, 9% of Australians reported delaying or forgoing a visit to a general practitioner or necessary prescription medications due to cost concerns,^5^ TB has been shown to exacerbate poverty,^6^ and although the relationship between TB and socioeconomics is understudied in Australia and other high-resource settings, research shows links between life events, such as personal or household illness, and lower perceived financial wellbeing.^3^

Victoria, Australia’s second-smallest state, has the country’s second-largest population and second-highest TB incidence rate. TB treatment is provided in specialist clinical settings, with the centralised Victorian Tuberculosis Program overseeing the public health response. Once a diagnosis of TB is made, a surveillance notification is sent to the Department of Health, and the Victorian Tuberculosis Program assigns a nurse for case and contact management. Identifying and addressing barriers to care for those affected by TB is a key component of the nurse's activities. TB-specific funding provides vouchers and small grants to offer financial relief for basic needs, such as food, communications, and transport. This assistance is granted based on assessed need during treatment and is not provided universally. Subsequently, the program meets all people diagnosed with TB disease in Victoria, serving as a unit of analysis for this study.

Initiated to facilitate the conceptualisation of program activity, the project informs practitioners and funders on intervention fidelity. Results influence practice through reflective and reflexive improvements. Whilst prospecting for fidelity (silver), patterns and trends (gold) were discovered and are described here.

## METHODS

Data^7,8^ and process mining^9,10^ approaches were adopted to enhance reflexive practice and provide meaning. The project began with prospecting potential data sources related to service recipients, financial relief and TB transmission, diagnosis and treatment. Financial relief dissemination records are routinely documented for budget accuracy and accountability. Like the routine public health data collected on case and contact management, TB characteristics, action and outcome, its informative value is greater than the raw data alone when systematic inquiry is made. These data have been examined separately or over shorter timeframes to enhance operational and programmatic decision-making. Consistent with arguments on the importance of monitoring overall care,^11,12^ the analysis plan was guided by the TB cascade of care framework.

We retrieved data for episodes of TB care and financial relief dissemination in Victoria between 1 January 2015 to 31 December 2023. Data were aggregated and systematically analysed using R (version 4.5.1), with queries guided by the TB cascade of care, characteristics of TB, and its human host. Measures of frequency, proportion, and central tendency were followed by exploration of evidence for associations with the occurrence of fund disbursement and demographic attributes, clinical features, planned and actual treatment duration, local transmission, linkage with healthcare, treatment and treatment outcome.

The grouping of genetically related isolates using a threshold of ≤5 single nucleotide polymorphisms (SNPs) by whole genome sequencing (WGS) to form clusters acts as markers of local transmission. Routine WGS has been completed for all eligible isolates since 2018 (inclusive). WGS was subject to request in 2015–2016 and funding through 2017, resulting in WGS completion for 58% of TB notifications within the study period. Given the exploratory nature of analysis and potential for collinearity, no statistical tests for significance were performed. Consistent with methodological best practice, practitioner documentation and consultation informed queries of the data and provide contextual meaning for each discovery.

### Ethical statement

Ethical approval for this project was provided by Melbourne Health (HREC 2022.179 RMH 90555).

## RESULTS

A total of 3,811 notifications of a TB diagnosis were received during the study period. TB-specific financial relief was provided to 15% of people. Compared to the broader cohort on treatment, those receiving financial relief were younger and more likely to be men (54% v 57%). They were more likely to have pulmonary disease (41 v 48%) and cavitation (9% v 17%). Treatment periods were often extended, and in cases where the extension was less than two months, case manager documentation and consultation indicated multiple brief interruptions. Documentation of treatment interruptions of greater than 2 months is mandated, and interruptions of this duration were 6.3 times more likely among those receiving financial relief. Incomplete treatment was 2.1 times more likely. Those receiving financial relief experienced shorter health care system delays. Significantly, those in receipt of TB-specific financial help were twice as likely to host *Mycobacterium tuberculosis* (*M.tb*) that clustered on WGS – see Table.

**Table.** Demographic and Clinical Characteristics by Receipt of TB-specific financial help.

Characteristic	No TB-specific financial help	TB-specific financial help	Total
	N (%)	N (%)	N (%)
Notifications	3226 (85)	585 (15)	3811 (100)
Men	1739 (54)	331 (57)	2070 (54)
People with pulmonary TB	1331 (41)	280 (48)	1611 (42)
People with pulmonary TB and cavitation	305 (9)	102 (17)	407 (11)
Documented treatment interrupted for 2 months or more (treatment completed)	7 (0.2)	8 (1.47)	15 (0.4)
Incomplete treatment*	64 (2)	24 (4)	88 (2)
Culture positive	2329 (72)	492 (84)	2812 (74)
With completed WGS	1825 (57)	398 (64)	2223 (58)
Of all cases identified as hosting WGS clustered TB (<6SNPS)	267 (8)	107 (18)	374 (10)
Proportion of those with WGS hosting clustered TB (<6SNPS)	(15)	(27)	
	Median (IQR)	Median (IQR)	Total Range
Age (years)	36 (27-58)	31 (24-43)	0-100
Treatment length in days	196 (183-275)	272 (186-327)	0-1957
Time between reported symptom onset and healthcare presentation (days)	24 (0-72)	25 (0-63)	0-3681
Time between healthcare presentation and treatment start delay (days)	39 (12-89)	17 (4-52)	0-8377

* Those lost to follow-up + those who did not complete treatment: <1% were still on treatment at the conclusion of the study period and are not represented here. Data extraction 28 February 2025

WGS = whole genome sequencing; SNP = single nucleotide polymorphisms; IQR = interquartile ranges.

Practitioner consultation on financial relief dissemination highlights the impact of marginalisation secondary to intersectionality. Practitioners speculate about delays in diagnosis and increased illness associated with shorter health care system delays. Further inquiry highlights the options available to, and behaviours of, those in this subgroup. This discussion is echoed in considering the distribution of financial relief to those with *M.tb* that is part of a WGS cluster. Those individuals are typically described as sharing traits across ethnicity, gender, age, wealth, income and employment. Disadvantage and vulnerability are observed in relation to key livelihood assets, including financial resources, housing, social networks, and health literacy.

The knowledge discovery provoked a discussion on the use of financial relief to mitigate economic barriers to care, as well as its role as an incentive or reward. Financial relief dispersal is based on assessed need. Most often, the need is documented as one of perceived or actual financial hardship. Vouchers also serve as external motivators, complementing advocacy efforts and systemic responses to assessed vulnerabilities and public health risks. Practitioners conclude that, regardless of whether financial relief is provided as an enabler, reward, or incentive, it must ultimately be beneficial and motivating for the recipient. Emphasised is the need to bridge the gap between diagnosis and treatment engagement.

## DISCUSSION

Of the 3,811 TB notifications, 15% received financial relief. Recipients were younger, more likely to be male, and pulmonary disease and cavitation were more frequent. TB treatment dynamics and transmission patterns were different for those who received financial relief. Combined with observations of access differences, our study has public health implications. The findings indicate a link between financial relief and both TB acquisition and care-related risk. Younger men, with more severe and transmissible forms of TB, are more likely to receive financial support – whether as relief, enabler or incentive – suggesting that need is shaped by intersecting vulnerabilities. Associations between financial relief and treatment linkage and engagement point to barriers in accessing care. Treatment interruptions raise concerns about increased opportunities for the development of *M.tb* that is drug-resistant. The findings of disadvantaged socioeconomic position, increased risk of TB acquisition and progression, correspond with Wu et al.’s^13^ definition of TB vulnerability.

Aligning with earlier studies, the need for an assessment for the dispersal of vouchers and grants suggests that some individuals with TB disease may experience disparities across multiple stages of care.^11,12^ Financial relief in the form of enablers closes the gap on out-of-pocket expenses, providing additional real choice beyond food *or* medication collection. In this setting, they also act as external motivators, giving practitioners time to understand non-monetary liabilities that have contributed to risk across the TB cascade of care and assets that can be leveraged, given sufficient assistance, e.g. applications for social protection payments.

Evident in the practitioner consultation and geospatial mapping of TB incidence in Victoria are intersectionality and structural vulnerability. This includes those described as key social determinants of TB – housing security, (built) environment, financial position, and culture.^6^ Observations made on the use of financial relief as an enabler or incentive align with understandings of how economic inequality shapes human behaviour. The description of those with TB that clustered on WGS as sharing traits consistent with economic disadvantage reinforces this, and is suggestive of Gobel and Carvacho’s^14^ argument of mutual constitution, in which behaviour shapes the culture of economic disadvantage. In Victoria, household transmission is implicated in more clusters identified through WGS, although a quarter of local transmission occurs in social or religious settings.^15^ Social and religious settings are shaped by social cohesion and cultural practices. This framing highlights the potential for financial and social interventions that can impact not just individual outcomes but community-level transmission.

The receipt of TB-specific financial relief is reported as favourably impacting programmatic goals of treatment engagement and completion, and reducing the burden of TB for those affected, particularly in high TB incidence settings.^16,17^ In settings with low TB incidence and high national income, data is limited; however, TB-specific financial relief has been associated with an increased likelihood of treatment completion.^16,18^ In our setting, financial relief complements advocacy, service navigation support, and system-level responses. Based on a perceived benefit in the context of vulnerability and hardship, financial relief cannot be separated from the relationship between the service provider and recipient. Practitioner consensus emphasises that funds must be meaningful to recipients. Our study supports the provision of financial relief, both to alleviate the material strain of out-of-pocket expenses associated with TB diagnosis and care, and as a source of motivation to link with TB care and engage with treatment. Reinforced is the need for strong linkage strategies to bridge the diagnosis, care and recovery gaps. Most significant is the observation of local TB transmission and its association with socioeconomic disparity and disadvantage.

The influence of psychosocial factors and marginalisation on TB is well documented globally,^19–21^ as is the broader impact of inequities.^14,22^ To further reduce the TB burden in Victoria (and similar settings), future research should prioritise capturing these contextual factors at the local level. Such research would deepen understanding of the contributors to TB vulnerability and how they interact. The knowledge generated from this study offers valuable insights and highlights opportunities to identify and address factors amenable to intervention.

Consistent with the methodology, the study has some limitations.^23^ Patterns are observed without inference of causal relationships. Data is impacted by veracity and available culture for WGS. Under-documentation and changing definitions of indicators (e.g. migration status, income and food security, homelessness) and access to care markers (e.g. ability to reach, pay, and engage)^13^ may understate actual levels of vulnerability. Of note is the timing of the work. The programmatic practice changes^24^ and livelihood protections associated with Victoria’s public health response to SARS-COV-2, which included 262 days of stay-at-home orders,^25,^ impacted the study period. The SARS-COV-2 public health measures and accompanying economic protections influenced the TB care cascade. The state’s social and economic recovery will be prominent in the post-study period. Prospecting existing data means applying retrieval criteria and accounting for practice changes. This can result in data fray at the date condition; however, for this project, the small number is unlikely to have a significant bearing on findings. Change and the possibility of fool’s gold are mitigated by the formative consultations with practitioners, which give context and history to inform meaning-making. This approach was selected to generate insights on the dissemination of financial relief and TB for program fidelity and to enhance decision-making through longitudinal data analysis. The method acknowledges that practitioners both consume and contribute to the generation and application of knowledge. Adopting a position of respectful curiosity, the project became an example of Epstein’s mining for silver and finding gold.^7^ Assessed financial need is implied in local TB transmission, disease progression, healthcare access and drug resistance risk. Further investigation of this vein gives utility for practice^23^ at the individual and household level, with the possibility of impacting the healthcare system and policy, mitigating structural inequities.
